# Balance impairment and cognitive dysfunction in patients with chronic obstructive pulmonary disease under 65 years

**DOI:** 10.1111/crj.13469

**Published:** 2022-01-26

**Authors:** Melike Hanim Kaygusuz, Ozge Oral Tapan, Utku Tapan, Sebahat Genc

**Affiliations:** ^1^ Department of Pulmonology Mugla Sitki Kocman University Mugla Turkey

**Keywords:** balance, cognitive function, COPD, dyspnea

## Abstract

**Objective:**

Balance and cognitive problems may develop in COPD. Studies investigating cognitive functions are mostly in elderly patients, and the number of studies on balance impairment is less than studies about cognitive dysfunction in COPD. We aimed to evaluate both balance and cognitive functions in patients with COPD under 65 years.

**Methods:**

A total of 60 COPD patients and 60 healthy control group were enrolled. The patients with COPD were divided into two groups due to dyspnea severity. Demographic data and dyspnea scores of all patients were recorded. BECK depression scale, standardized Mini Mental Test (MMT), and BERG balance scale were applied to the individuals. Factors affecting BERG balance scale were evaluated.

**Results:**

BERG balance scale and MMT values were found to be significantly lower in patients with COPD compared with the control group (*p* = 0.009). It was observed that the mean values of BERG balance scale and MMT were significantly low in the severe dyspnea group. BERG scale had significant correlations with MMT (rho = 0.331, *p* = 0.001), BECK (rho = −0.222, *p* = 0.016), age (rho = −0.318, *p* = 0.018), MMRC (rho = −0.368, *p* < 0.001), CAT (rho = −0.338, *p* = 0.008), FEV1% (rho = 0.307, *p* = 0.017), SpO_2_ (rho = 0.448, *p* < 0.001), and number of hospitalization per year (rho = −0.316, *p* = 0.014). According to the results of multiple linear regression analysis, the effect of oxygen saturation on BERG was found to be statistically significant (B = 0.512, *p* = 0.008).

**Conclusions:**

Balance tests in patients with COPD under the age of 65 are significantly low when compared with healthy controls at the same age. Balance and cognitive functions are significantly associated with each other. It may be beneficial to perform balance and cognitive function tests earlier even at the beginning of the treatment period of COPD, and strategies should be planned to prevent the progression of functional losses.

## 1 INTRODUCTION

Chronic obstructive pulmonary disease (COPD) is a common, preventable, and treatable disease that is characterized by persistent respiratory symptoms and airflow limitation.^1^ At the most severe stage of COPD, quality of life is significantly reduced because of ongoing shortness of breath. Trouble breathing may even be life‐threatening during some episodes. The most important goal of COPD treatment is to improve the quality of life. Dyspnea in COPD patients leads to impaired physical coordination and muscle loss in the long term. These result in a feeling of fatigue and more dyspnea. Increased fatigue and dyspnea with disease progression in COPD complicates daily life activities. Loss of functional capacity and life quality may rise the anxiety of patients with COPD.^2^ Anxiety and depression are reported to be the most common mental disorders associated with COPD.^3^, ^4^

Cognitive impairment has been reported in cases with hypoxic COPD.^5^, ^6^, ^7^ Cognitive problems may occur in severe stages of COPD.^8^ Acute, chronic, and systemic inflammation, night desaturations, and hypercapnia have been associated with cognitive impairment.

Cognitive impairment results with both decrease in the quality of life and discordance to physical exercise programs. Depends on verbal memory loss, a decrease in inhaler treatment compliance and an increase in the number of exacerbations were reported.^9^

Decrease in mechanoreceptors in the knee joint with age causes loss of balance and proprioception.^10^ Several articles have shown that balance is negatively affected in patients with COPD and it has been reported that the frequency of falling is increased.^11^, ^12^, ^13^ Falls increase morbidity and mortality in patients with COPD.^14^, ^15^ Muscle weakness, vision disorders, side effects of drugs, depression, and cognitive disorders are the factors causing the falls.^16^, ^17^

The contributing factors for balance impairment in COPD may be reductions in proprioceptive control, trunk and respiratory muscle coordination, skeletal muscle dysfunction, reduced exercise capacity and physical activity levels, dyspnea, and systemic inflammation.^18^, ^19^, ^20^, ^21^, ^22^, ^23^, ^24^, ^25^, ^26^ Balance impairment following an acute exacerbation of COPD is more often.^20^, ^27^ Studies investigating balance impairment in COPD are limited, and the studies on cognitive functions in COPD are mostly in elderly patients. In this study, our aim was to evaluate the balance and cognitive impairments in patients with COPD under 65 years. We tried to find out the clinical factors, which may be related with balance impairment.

## 2 MATERIALS AND METHODS

Study approval was obtained from the Clinical Research Ethics Committee of Mugla Sitki Kocman University Faculty of Medicine. After their informed consent is obtained, patients with COPD less than 65 years were enrolled to the study between September 2019 and March 2020. The sample size was calculated with the G‐power program. Sixty total COPD patients and 60 healthy control group were included in the study. COPD patients who were diagnosed with any psychiatric or neurological diseases and orthopedic problems that will affect compliance with the balance test were excluded. Those with a score of >19 on the BECK depression scale in the healthy volunteer group were excluded from the study because depression may affect MMT.

Since dyspnea in COPD patients leads to impaired physical coordination and muscle loss in the long term, the patients were divided into two groups due to dyspnea severity and compared with the active smoker control group without dyspnea. Dyspnea severity was assessed by Modified Medical Research Council (MMRC) Dyspnea Scale ≥2. Controls were the volunteers who had admissions to our smoking cessation clinic. Controls did not have any diagnosed chronic pulmonary disease. Demographic data and exacerbation history were collected. BECK depression scale, standardized Mini Mental Test (MMT), and BERG balance scale were applied to both COPD patients and control group. The CAT and MMRC dyspnea scores of COPD patients were recorded.

MMT is a commonly used cognitive function test that includes skill questions for determining the orientation, attention, memory, language, and visual–spatial in adult patients. The maximum possible score is 30 points. With points between 25 and 20 shows decreased cognitive functions and a score of <20 is considered as marked cognitive impairment in individuals. The same test is available for the uneducated people.^28^ Both versions were used in our study. The BERG balance scale directly observes performance, balance, and risk of falling. It is a 14‐item scale that evaluates quantitatively. Completion of the scale and statically measurement of the patient's ability to maintain balance requires 10 to 20 min depending on its performance.

An overall score is calculated from the 56 possible points. Scores of 0 to 20 points are described as “balance disorder”; scores of 21 to 40 are interpreted as “acceptable balance”; and scores of 41 to 56 are generally classified as “good balance.” With this scale, both static and dynamic balance can be evaluated.^29^

BECK depression scale measures the severity of depression in adolescents and adults. It is a widely used 21‐item self‐report inventory. In this scale, patients are asked to respond according to their mood for the last 2 weeks. It is widely used as an indicator of the severity of depression but is not a diagnostic tool. The score of the test varies between 0 and 63. Results 0–9 are evaluated as none or minimal depression, 10–18 as mild depression, 19–29 as moderate depression, and 30–63 as severe depression.^30^

MMRC scale was modified by the British Medical Research Board and started to be used in lung diseases. This is a five‐item scale that is used to classify dyspnea from the mild to severe during various physical activities.

The COPD Assessment Test measures the quality of life of COPD patients. It consists of eight questions that show the degree of exposure to the disease in the best way.

### 2.1 Statistical analysis

Statistical analysis of the data of our study was made by using SPSS 21.0 package program. Descriptive data were indicated with frequency, percentage, mean, and standard deviation. To determine whether the data conform to the normal distribution, Kolmogorov–Smirnov Test was applied. Student's *t* test or Mann–Whitney *U* test were performed to test for significant differences between groups. To determine the relationship between variables, depending on whether the data are parametric or non‐parametric, Pearson or Spearman correlation analysis was used, and *p* < 0.05 value was accepted as significant for statistical analysis. Multivariable regression was performed for the variables that showed a significant association.

## 3 RESULTS

A total of 120 people, 60 of whom were diagnosed as COPD and 60 as healthy volunteers, were enrolled to our study. Demographic data of the participants were summarized in Table 1. Male gender was significantly high in COPD patients. There was no significant difference in education between groups.

**TABLE 1:** Demographic data, BERG, and MMT scores of COPD patients

	Group 1 (mean ± SD) *n* = 30	Group 2 (mean ± SD) *n* = 30	*p*
Age (years)	55.00 ± 7.96	59.33 ± 4.22	**0.015** ^b^
Gender (female/male)	1/29	1/29	0.754^c^
Education (≤Primary school/≥Middle school)	18/12	16/14	0.894^c^
Smoking (active smoker/ex‐smoker)	14/16	12/18	0.915^c^
Pack‐year (years)	33.90 ± 17.83	47.98 ± 36.48	0.062^b^
BMI	26.57 ± 4.34	25.66 ± 5.32	0.228^a^
MMRC	0.60 ± 0.49	2.97 ± 0.76	**<0.001** ^b^
CAT	4.37 ± 2.77	23.63 ± 6.95	**<0.001** ^b^
FEV1 (%)	48.53 ± 16.33	38.60 ± 13.76	**0.014** ^a^
FVC (%)	63.97 ± 18.10	54.13 ± 16.20	**0.031** ^a^
SpO_2_ (%)	94.73 ± 3.95	91.60 ± 3.24	**<0.001** ^b^
Annual exacerbation (*n*)	0.87 ± 2.14	4.80 ± 5.27	**<0.001** ^b^
Number of hospitalization (*n*/year)	0.13 ± 0.34	1.43 ± 1.71	**<0.001** ^b^
BERG	55.20 ± 1.62	52.97 ± 4.50	**0.028** ^b^
MMT	26.63 ± 2.94	25.97 ± 3.36	0.452^b^
BECK	4.27 ± 3.34	7.20 ± 4.50	**0.006** ^a^

*Note*: Group 1: MMRC <2. Group 2: MMRC ≥2. Bold emphasis was used to draw attention for the statistically significant results.

Abbreviations: BMI, body mass index; CAT, COPD Assessment Test; FEV1, forced expiratory volume in 1 s; FVC, forced vital capacity; MMT, Mini Mental Test; MMRC, Modified Medical Research Council; SpO_2_, oxygen saturation.

^a^ *T* test.

^b^ Mann–Whitney *U* test.

^c^ Chi‐square.

The mean packet year of the COPD patients with high CAT and MMRC scores was significantly higher. The mean age, annual number of exacerbations, and number of hospitalizations of the patients in the COPD patients with severe dyspnea were significantly higher while the values of FEV1 (%), FVC (%), oxygen saturation (SpO_2_), and BERG balance score were found to be lower (Table 1). However, there was no significant difference in the mean MMT value of COPD patients (Table 1). The mean age of the control group was 56.03 ± 5.53, and the education status was similar to the COPD group. Body mass index (BMI), SpO_2_, BERG, and MMT scores were significantly high in control group (Table 2). BECK depression score was significantly low in the control group (Table 2). BECK values of the COPD patients with severe dyspnea were found to be significantly higher (Table 1). BECK depression score had significant correlations between FEV1% (rho = −0.355, *p* = 0.005), MMRC (rho = 0.497, *p* < 0.001), SpO_2_ (rho = −0.342, *p* = 0.001), BERG (rho = −0.222, *p* = 0.035), MMT (rho = −0.253, *p* = 0.016), annual exacerbation (rho = 0.357, *p* = 0.005), and number of hospitalization per year (rho = 0.287, *p* = 0.026). It was observed that the BERG balance scale scores of the group with SPO_2_ < 90% in the COPD patient population were significantly lower (*p* = 0.03).

**TABLE 2:** Demographic data BERG and MMT scores of individuals

	COPD patients (mean ± SD) *n* = 60	Control group (mean ± SD) *n* = 60	*p*
Age (years)	57.17 ± 6.69	56.03 ± 5.53	0.314^b^
Gender (female/male)	2/58	22/38	**<0.001** ^c^
Education (≤Primary school/≥Middle school)	34/26	21/39	**0.017** ^c^
Smoking (active smoker/ex‐smoker)	26/34	51/9	**0.432** ^c^
Pack‐year (years)	40.94 ± 29.34	22.38 ± 8.53	**<0.001** ^b^
BMI	26.12 ± 4.83	27.71 ± 3.76	**0.047** ^a^
SpO_2_ (%)	93.17 ± 3.91	98.10 ± 0.89	**<0.001** ^b^
BERG	54.08 ± 3.54	55.80 ± 0.40	**0.009** ^b^
MMT	26.30 ± 3.15	29.63 ± 0.74	**<0.001** ^b^
BECK	5.73 ± 4.20	4.33 ± 3.22	**0.043** ^a^

*Note*: Bold emphasis was used to draw attention for the statistically significant results.

Abbreviations: BMI, body mass index; MMT, Mini Mental Test; SpO_2_, oxygen saturation.

^a^ *T* test.

^b^ Mann–Whitney *U* test.

^c^ Chi‐square.

**FIGURE 1:**
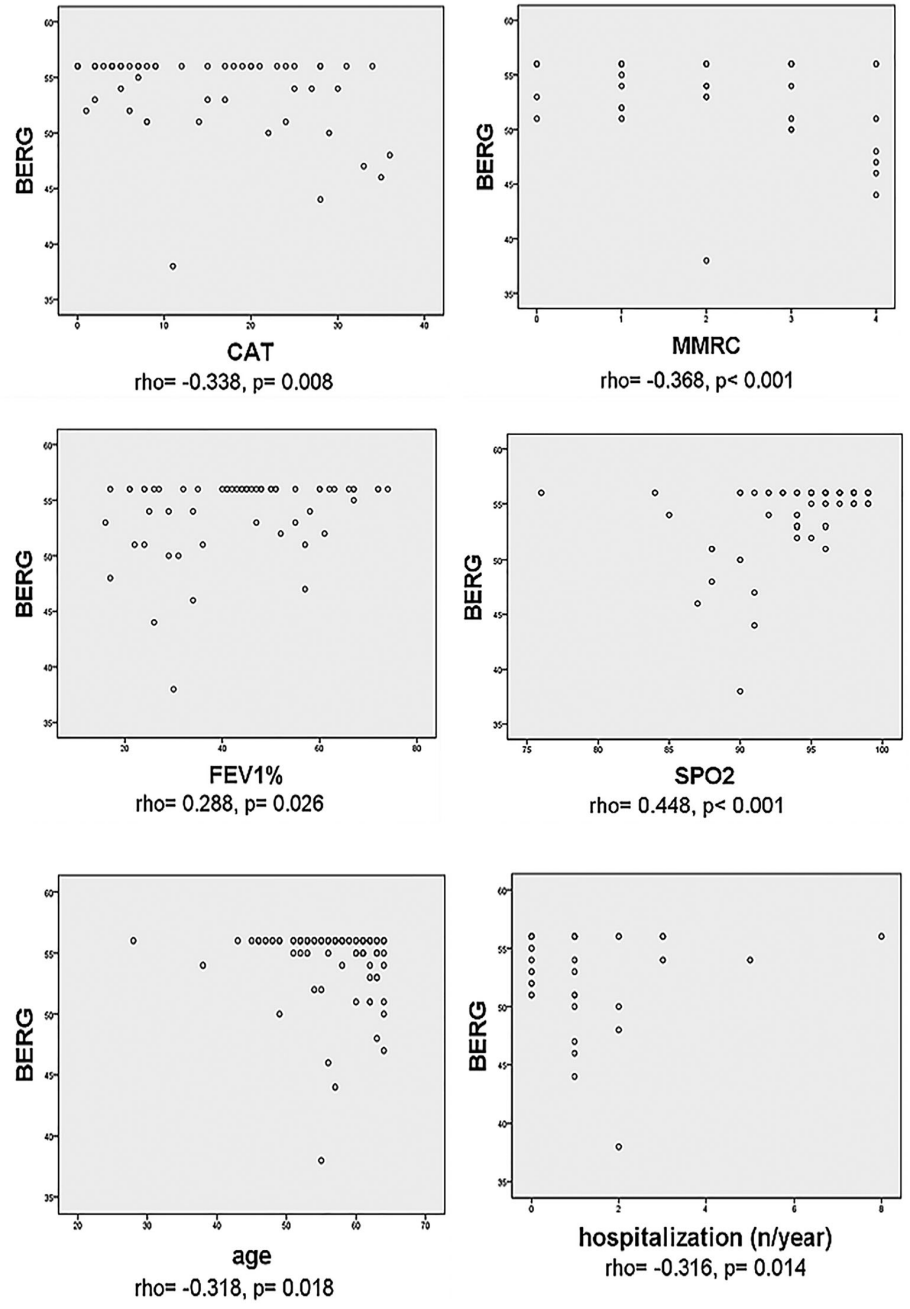
Scatter plots between BERG and related variables

Spearman correlation analysis was performed to determine the relationships between MMT, BERG values, and characteristic factors of COPD patients. There was a weak (rho = 0.331) and statistically significant relationship (*p* = 0.001) between MMT and BERG. A significant relationship was found between MMT and BERG as a result of simple linear regression analysis (B = 0.314, SE = 0.099, 95% CI = 0.117–0.512, *p* = 0.002). Significant correlations were calculated with age, smoking pack‐year, MMRC, CAT, FEV1, SpO_2_, and number of hospitalization (Table 3). However, the only significant variable associated with BERG was SpO_2_ (B = 0.512, SE = 0.185, 95% CI = 0.139–0.884, *p* = 0.008) in multivariable regression analysis. The relationships between BERG and the variables were shown in Figure 1.

**TABLE 3:** Relationships between MMT, BERG, and characteristics of COPD patients

			MMT	BERG
Spearman's rho	MMRC	Rho	−0.446	−0.368
		*p*	**<0.001**	**<0.001**
	CAT	Rho	−0.139	−0.338
		*p*	0.290	**0.008**
	FEV1 (%)	Rho	0.122	0.288
		*p*	0.351	**0.026**
	SPO_2_	Rho	0.551	0.448
		*p*	**<0.001**	**<0.001**
	Hospitalization (*n*/year)	Rho	−0.130	−0.316
		*p*	0.323	**0.014**
	Age (year)	Rho	−0.248	−0.318
		*p*	**0.018**	**0.018**
	Pack‐year (*n*)	Rho	−0.401	−0.053
		*p*	**<0.001**	0.618

*Note*: Bold emphasis was used to draw attention for the statistically significant results.

Abbreviation: MMT, Mini Mental Test.

## 4 DISCUSSION

Our findings show that balance tests in patients with COPD under the age of 65 are significantly low when compared with healthy controls at the same age. Balance and cognitive functions are significantly associated with each other. FEV1 value, cognitive functions, oxygen saturation, dyspnea severity that was evaluated with MMRC and CAT have significant correlations with balance. However, the only significant factor affecting BERG was the oxygen saturation.

COPD is a multicomponent disease associated with multiple comorbidities, social and psychological disorders. Balance and cognitive impairments were associated with decreased quality of life and increased mortality in the general population.

Chronic diseases require optimum management of the situation, as well as learning new skills: basic personal care functions and the ability to initiate, organize, and conduct daily treatment. Impairment occurring in the nervous system with older age affects cognitive functions, balance and perceptual abilities which are important for seeing obstacles and other necessary motor skills. These disabilities may be seen in early ages among patients with COPD when compared with healthy volunteers at the same age. The prevalence of cognitive impairment was reported as 32.8% in COPD.^31^ Martin et al.^32^ declared the decrease in cognitive function caused an increase in the frequency of falling in 386 adults between the ages of 60 and 86. Falls have been identified as a major public health problem, and they are the third leading cause of accidental deaths at all ages. Twenty‐eight percent of traumatic brain injury (TBI) cases are from falls.^33^ Falls cause a decrease in quality of life and are frequent in patients with COPD, but risk of falling seems less important than the respiratory consequences of the disease. Hospital admissions due to falls increase the total cost of COPD. In our study, there was a statistically significant positive relationship between cognitive impairment and balance disorders.

Studies on cognitive functions in COPD have been conducted on hypoxemia. Chronic hypoxia creates neuronal damage and loss. Night desaturations and hypercapnia also affect cognitive functions.^34^, ^35^, ^36^ Liesker et al.^7^ detected cognitive dysfunction in also non‐hypoxemic COPD patients. The researchers thought it might be related to desaturations at night and decreased health‐related quality of life. In our study, loss of cognitive functions was statistically significant in the COPD patients compared with healthy volunteers at the same age. COPD patients with low MMRC had higher MMT values. MMT is a measure for severe dementia, and it is insensitive for mild cognitive impairment.^37^ Therefore, it could not predict mild cognitive dysfunction. This is one of the limitations of our study. The relationships between cognitive dysfunction, age, and education have been studied before. It has been shown that the prevalence of cognitive impairment decreased from 32.9% to 15.5% with a 3‐year increase in education time.^38^ In our study, education levels of the COPD patients and the control group were similar. This result eliminated the effect of education level on cognitive tests.

Roig et al.^17^ studied cardiovascular diseases as one of the possible causes of the increased frequency of falls in patients with COPD. Cardiac and anti‐hypertensive drugs cause postural orthostatic hypotension and increasing syncope attacks. Corticosteroids reduce the visual functions and increase the risk of osteoporosis. The depressants frequently increase the reaction time to events. Reduced attention may increase the risk of falling. In addition, malnutrition and decreased muscle strength and endurance and atrophy due to decreased activity are predicted to increase the loss of balance. Muscle mass loss in people with COPD is associated with increased mortality, impaired muscle function, decreased exercise capacity, and poor quality of life.^13^ Mathur et al.^39^ compared muscle volume of patients with COPD to a healthy control group. They reported 25% reduction in strength and about 35% greater intramuscular fat in COPD group. Physiological changes due to aging, physical inactivity, and comorbidities worsen postural control and increase the fall risk in COPD.^40^ In our study, it was observed that the mean BERG score of the patients with severe dyspnea were significantly lower. Our results speculate that dyspnea severity measured with MMRC affect the balance of COPD patients negatively whereas high FEV1 and SaO_2_ have positive effects on the balance these patients. These results may depend on the emphysema and decreased physical activity due to breathlessness which leads to muscle wasting. A reduction in calorie intake has been reported as one of the potential mechanisms leading to a catabolic state in COPD.^41^ In our study, the negative correlation between BERG and the number of hospitalization for acute exacerbations per year supports that the catabolic effects of exacerbations worsen the muscle strength. But we did not measure the muscle strength and exercise capacity of the patients. This is a limitation of our study.

Although balance impairment in COPD is a condition that should be carefully monitored, routine assessment is still not a part of international guidelines. The use of balance outcomes was commented by American Thoracic Society/European Respiratory Society,^42^ and treatment of balance impairment is becoming more important within pulmonary rehabilitation.^43^

## 5 CONCLUSION

Considering that impairments of balance and cognitive function increase with age, early examination, and close follow up of COPD patients are thought to be important for early diagnosis and treatment of functional losses. We recommend focusing on pulmonary rehabilitation to prevent balance loss. Also, the depression level and cognitive function that are related to balance impairment for COPD patients have to be evaluated.

## CONFLICT OF INTEREST

The authors declare that they have no competing interests.

## AUTHOR CONTRIBUTIONS

Conception and design: Ozge Oral Tapan, Melike Hanim Kaygusuz. Administrative support: Melike Hanim Kaygusuz. Provision of study materials or patients: Ozge Oral Tapan, Utku Tapan, Sebahat Genc. Collection and assembly of data: Ozge Oral Tapan, Melike Hanim Kaygusuz. Data analysis and interpretation: Ozge Oral Tapan, Melike Hanim Kaygusuz. Manuscript writing: Ozge Oral Tapan, Melike Hanim Kaygusuz. Final approval of manuscript: Ozge Oral Tapan, Melike Hanim Kaygusuz, Utku Tapan, Sebahat Genc.

## ETHICS STATEMENT

All included patients had signed written consent form, and approval was obtained from Muğla Sıtkı Koçman University Faculty of Medicine Clinical Research Ethics Committee with its decision number 09/III.

## Data Availability

Individual‐level data used for this study are held within the servers of the Mugla Sitki Kocman University, Faculty of Medicine, Department of Pulmonology, and could not be publicly available due to privacy reasons. Thus, the datasets generated and/or analyzed during the current study are not publicly available due to confidentiality agreement but are available from the corresponding author on reasonable request as long as the request meets the ethics.
